# Trends in the burden of varicella in UK general practice

**DOI:** 10.1017/S0950268817001649

**Published:** 2017-08-30

**Authors:** J. L. WALKER, N. J. ANDREWS, R. MATHUR, L. SMEETH, S. L. THOMAS

**Affiliations:** 1 Statistics, Modelling and Economics Department, Public Health England, 61 Colindale Avenue, London NW9 5EQ, UK; 2 Faculty of Epidemiology & Population Health, London School of Hygiene & Tropical Medicine, Keppel Street, London WC1E 7HT, UK

**Keywords:** Electronic health records, primary healthcare, UK, varicella

## Abstract

Childhood varicella vaccination has not yet been introduced in the UK. To inform decision-making about future vaccine programmes, data on the burden of varicella in general practice over a 10-year period (01/01/2005–31/12/2014) was calculated by age and ethnicity, using anonymised data from >8 million individuals in the Clinical Practice Research Datalink. Varicella consultations peaked at 20 603 in 2007, then decreased annually in all age groups to 11 243 in 2014. Each year, consultation rates were common among infants, were highest among 1–3 year olds (61·2 consultations/1000 person-years in 2007, 39·7/1000 person-years in 2014) and then fell with increasing age to <1·0/1000 person-years at ages ⩾20 years. Varicella acquisition appeared to be delayed in some ethnic groups, with lower consultation rates for children aged <3 years but increased rates for older children and adults aged ⩽40 years among those of black African, Afro-Caribbean, South Asian or other Asian ethnicity. Decreasing general practice consultation rates over time could reflect changes in healthcare utilisation, with patients seeking care in alternative settings such as Accident and Emergency Departments, although current data prevent full assessment of this. Availability of data on varicella diagnoses across all health settings would enable estimation of the total healthcare burden due to varicella and the cost-effectiveness of introducing varicella vaccination.

Varicella remains a common disease of childhood in temperate countries such as the UK that do not have universal childhood varicella vaccination programmes. In these countries, most individuals have acquired varicella-zoster-virus infection by the age of 15 years [1]. In contrast, in some tropical climates, the average age at infection is later, with an appreciable proportion of the population remaining susceptible in early adulthood [2]. Varicella is typically a mild self-limiting disease in healthy children but complications are more common in adulthood. Thus, migrants from specific regions who move to temperate countries after childhood can be at particular risk of acquiring varicella as adults, with the risk of developing more severe disease.

In England, varicella vaccination is offered currently only to non-immune healthcare workers and to household contacts of immunosuppressed individuals [3]. In 2009, the UK Joint Committee for Vaccination and Immunisation assessed the clinical, epidemiological and economic evidence to inform decisions about possible vaccination strategies against varicella and concluded that a childhood varicella programme would not be cost-effective [4]. However, it was agreed that this recommendation would be kept under review as new data on the epidemiology of varicella and herpes zoster became available.

As varicella is not a notifiable disease in the UK, multiple data sources are needed to assess its healthcare burden. Trends in hospitalisations for varicella in England between 2004 and 2013, assessed using inpatient Hospital Episode Statistics, were recently published and showed that hospitalisation rates were broadly stable [5]. Here, we report trends in the burden of varicella seen in general practice, by age and ethnicity.

The data source used was the Clinical Practice Research Datalink (CPRD), a large database of anonymised UK general practice records comprising a representative sample of approximately 7% of the UK population [6]. The study population comprised all patients registered with CPRD practices that met CPRD-defined data quality standards (‘up to standard’ (UTS) data) between 1 January 2005 and 31 December 2014. The start of follow-up for each individual was the latest of their registration date (if aged ⩽6 months at registration), 6 months after their registration date (for patients aged >6 months at registration, to exclude historical episodes of varicella recorded retrospectively when the patient joined the practice), the practice UTS date and 1 January 2005 [7]. Individuals’ end of follow-up was the earliest of the date they left the practice or died, the practice's last data collection date and 31 December 2014. General practice consultations for varicella were identified using Read codes and consultation codes that indicated a general practice contact, excluding consultations from other settings (e.g. hospitalisations).

Covariates of interest included age, calendar year and ethnicity. Age was initially categorised into <1, 1–3, 4–6, 7–10, 11–20 and >20 years, and then categorised more finely to explore age differences by ethnicity. Ethnicity was identified using the methods of Mathur *et al.*, with categorisation informed by countries of origin where there may be later average age at varicella, namely white, mixed, Caribbean, South Asian, other Asian, African/other black and other ethnicities [2,8].

In primary analyses, all varicella consultation-days occurring in the 12 months after a first-ever varicella consultation were included; the rationale was that, given the rarity of second episodes of varicella and the expected short duration of uncomplicated varicella, consultations occurring after the 12-month period in these analyses could be historical recording of past varicella or miscoded non-varicella events. In sensitivity analyses, consultations were (a) restricted to those occurring in the 6 months after an initial varicella consultation and (b) counted irrespective of varicella history, including all consultation-days over time. Poisson-distributed consultation rates with 95% confidence intervals (CIs) were calculated by age and calendar year, using CPRD person-time denominators. Ethnic-specific consultation rate analyses were restricted to 2006–2012, the years during which general practitioners (GPs) were financially incentivised to record patients’ ethnicity [9].

Approval for the study was obtained from the Independent Scientific Advisory Committee of the Medicines and Healthcare Products Regulatory Agency (ISAC number: 15_065) and from the Ethics Committee of the London School of Hygiene & Tropical Medicine (LSHTM reference: 12133).

There were 8 432 826 individuals recorded for at least 1 day in CPRD between 1 January 2005 and 31 December 2014. Median follow-up time was 4·9 years (interquartile range 1·8–9·3 years). During the study period, 151 716 individuals had a first-ever varicella event recorded. These individuals had 160 166 varicella consultations; 141 586 individuals (93·3%) had a single varicella consultation and 10 130 had one or more subsequent varicella consultation recorded 1 day to 9·6 years later. In total, 62% of those with multiple varicella consultations completed all subsequent consultations within 30 days of the initial consultation, and 76% completed their consultations within 12 months. Only 2711 consultations (1·7%) occurred more than 12 months after the first-ever varicella consultation and these were excluded from the primary analysis.

The number of consultations peaked in 2007 at 20 603 and then fell over time to a low of 11 243 in 2014. Age-specific varicella consultation rates for each year of the study period are summarised in Fig. 1*a*. Consultation rates declined over time across all age groups, with decreases ranging from 6% (in 4–6 years old) to 54% (in 20+ years old) over the entire study period. Consultations were common among infants, peaking at 41·5/1000 person-years in 2007, but the most frequent consultations were among children aged 1–3 years (61·2 consultations/1000 person-years in 2007, 39·7/1000 person-years in 2014). Rates fell successively with increasing age; in those aged 20+ years, the rates were less than one varicella consultation/1000 person-years throughout the study period. In the two sets of sensitivity analyses, the number of consultations and consultation rates were very similar to those in the main analysis (data not shown), reflecting the very large proportion of individuals who had only a single varicella record.

**Fig. 1.**
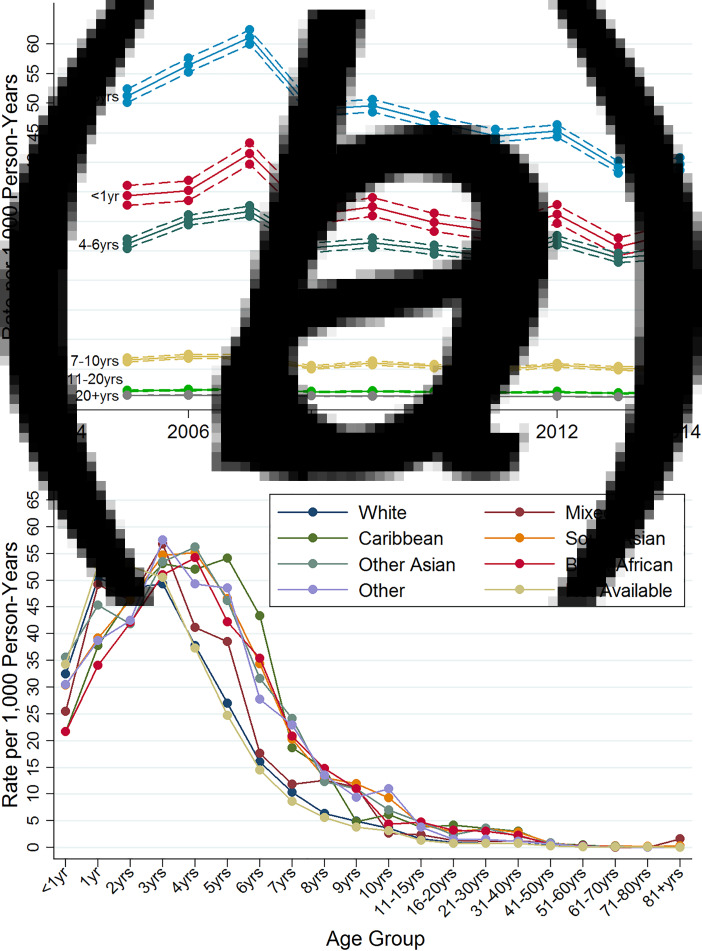
Varicella consultation rates: (*a*) with 95% confidence intervals (dotted lines) by age and year; (*b*) by age for each ethnic group (for the years 2006–2012).

Age-specific consultation rates for 2006–2012 for the seven ethnic groups, and for those with no ethnicity recorded, are shown in Fig. 1*b*. Completeness of recording of ethnicity varied from 61% (among those aged <1 year) to 34% (among those aged 80+ years). For those aged <16 years, there was a slight shift to older ages at consultation for several non-white ethnic groups, with lower rates below the age of 3 years but higher rates for older children. For example, children of black African and Afro-Caribbean origin had median ages at consultation of 4·2 and 4·5 years, respectively, compared with a median age of 2·8 years for children of white ethnicity. Among older children aged 11–15 years, black African children had nearly three times the consultation rates for varicella compared with those of white ethnicity (4·7/1000 person-years, 95% CI 3·7–5·7 *vs.* 1·6/1000 person-years, 95% CI 1·5–1·7), with similar higher rates among South Asian, other Asian and Afro-Caribbean children. These four non-white ethnic groups also had higher consulting rates (with non-overlapping 95% CIs) compared with those of white ethnicity at ages 16–20 years (2·3–4·2/1000 person-years *vs.* 1·0/1000 person-years), and at ages 21–30 and 31–40 years. Among those aged 41–50 years, rates were higher for individuals of other Asian and South Asian ethnic origin. Those with missing information on ethnic origin had similar consultation rates to those of white ethnic origin in all age groups. To explore further the potential impact of missing data, we repeated analyses restricted to 2012 (the year for which ethnicity was most completely recorded); results were almost identical to those obtained for 2006–2012 (data not shown).

We postulated that the decrease in general practice consultation rates over the study period could be due in part to changes in health-seeking behaviour. For example, patients with varicella may have presented increasingly over time to Accident and Emergency (A&E) departments directly rather than attending their GP as a result of changes in GP service provision, as has been suggested for other acute illnesses in the UK [10]. We therefore analysed the proportion of first varicella consultations over time that had a record on the same day indicating an A&E Department visit or a hospitalisation. We found no evidence of an increasing trend in varicella consultations in secondary care: for those aged ⩽16 years, 2·8% of all first varicella consultations in both 2005 and 2014 had a hospital visit recorded (with a peak of 3·2% in 2011). Similarly, for >16 years old, 2·2% of all first varicella consultations were in hospital or at A&E departments in 2005 compared with 2·8% in 2014 (peaking at 2·9% in 2010).

Our finding that the highest healthcare burden of varicella was among very young children is consistent with studies from an earlier era that showed changes in the age distribution of varicella cases presenting to general practice, with an increasing proportion of cases among those aged <5 years [11]. It is thought that this change reflects increasing daycare attendance over time by pre-school children in the UK. Although country of birth data are not well recorded in CPRD, our ethnicity-specific analyses are consistent with reports that age at varicella acquisition may be delayed in some tropical countries and that adults in some non-white ethnic groups are at increased risk of varicella [2]. However, overall consultation rates for varicella in adulthood remained low in these groups. Among children, the older age at varicella acquisition for several ethnic groups may also be due in part to being born outside the UK, but could also result from different mixing patterns in early childhood, for example, lower daycare attendance.

Our study included a very large study population that is broadly representative of the UK population and with data available over a long period. Some estimates of rates stratified by both age and ethnicity were based on small numbers, although non-overlapping confidence intervals for estimates suggested that differences in rates were unlikely to be due to random error. The ethnicity analyses were also limited by missing data. We did not classify those with no recorded ethnicity as being from a white ethnic group, an approach adopted by some researchers on the basis that, if the study population reflected the UK population, at least 93% of these individuals would be likely to be of white ethnicity [12]. However, the almost identical rates at every age for those of white and of unstated ethnicity does suggest that most of these individuals were indeed of white ethnic origin. With the removal of the contractual requirement to record patients’ ethnicity, the extent of missing data in general practice data may increase in future analyses, although the British Medical Association has suggested that practices would want to continue to record routinely patients’ ethnicity to enable assessment of the needs of their practice population [9].

Some other potential limitations need consideration. The outcome of interest was clinically diagnosed varicella without virological confirmation. However, varicella has a very characteristic clinical presentation and misdiagnosis by GPs is unlikely. Inclusion of all consultations in the 12 months after the first varicella record may have included some non-varicella consultations, for example, GP recording of past varicella or contacts with varicella, and this may have resulted in overestimates of consultation rates. However, we obtained near-identical consultation rates when we repeated analyses after truncation of later consultations to 6 months, and then when all varicella consultations were included with no restrictions. Conversely, removal of duplicate varicella consultations on the same day may have removed some genuine re-consultations for varicella, resulting in slight underestimation of rates; but given that varicella is generally a mild disease, it is likely that many of these excluded same-day consultations were duplicate recording (e.g. separate records for requesting tests, or referrals).

As severe varicella is uncommon in healthy children and uncomplicated varicella requires no specific treatment, many varicella cases do not present to general practice. Our study was not designed to estimate incidence of varicella, but rather to contribute to estimates of the healthcare burden of varicella in different settings to inform future cost-effectiveness analyses of any UK varicella vaccination programme. A decrease in varicella cases seen in general practice over time could be due to decreasing incidence of varicella in the community, a tendency for GPs to diagnose varicella less often when patients present with possible varicella, or changes in healthcare utilisation. The first two of these possible explanations seem unlikely. We did not find that the marked decrease in general practice consultations observed over the study period was mirrored by corresponding increases in the burden seen in other healthcare settings. Recent separate analyses of hospitalisation data in England have shown no notable increases in varicella hospitalisations over a similar time period [5]. However, we were only able to assess A&E attendance for varicella using general practice records, and it is likely that these attendances are under-recorded by GPs. Until very recently, Hospital Episode Statistics A&E data recorded only a small proportion of diagnoses made in A&E settings and so these data could not be used to identify varicella visits. Improvements to recording of varicella diagnoses in A&E data, and in other data sources such as calls to the NHS 111 phoneline, will enable future assessment of changes in health-seeking behaviour data and more complete data on varicella cases across healthcare settings. This will maximise assessment of the overall healthcare burden attributed to varicella and support decision-making about whether to introduce varicella vaccination in the UK.
